# In-Situ Pixel-wise Emissivity Measurement Using a Multispectral Infrared Camera

**DOI:** 10.3390/jimaging9100198

**Published:** 2023-09-27

**Authors:** Corentin Poissenot-Arrigoni, Bertrand Marcon, Frédéric Rossi, Guillaume Fromentin

**Affiliations:** Arts et Métiers Institute of Technology, LaBoMaP, UBFC, HESAM, F-71250 Cluny, France; bertrand.marcon@ensam.eu (B.M.); frederic.rossi@ensam.eu (F.R.); guillaume.fromentin@ensam.eu (G.F.)

**Keywords:** infrared thermography, emissivity, pixel-wise, multispectral camera

## Abstract

In the thermography process, accurately determining emissivity is crucial to obtain precise temperature measurements as it enables the conversion of radiometric values to absolute temperatures. However, assessing emissivity is not a straightforward task as it depends on various other parameters. Traditional methods for measuring emissivity often involve costly materials and cannot be carried out simultaneously with infrared image acquisition. This article presents a method for obtaining pixel-wise emissivity using data from a multispectral infrared camera. Consequently, this method allows for direct emissivity measurement during infrared camera acquisition without the need for additional materials or experiments.

## 1. Introduction

With the increasing accessibility of infrared cameras, thermography is being widely used in various fields. For example, it is employed in characterizing the thermal behavior of materials during mechanical tests such as tensile tests [1], fatigue tests [2], and machining operations [3]. Additionally, it is utilized in the detection of defective products [4,5,6,7,8], or to characterize materials [9,10,11,12]. This measurement technique is relatively simple to set up and is non-destructive. However, achieving accurate temperature measurements is not straightforward because of potential sources of error [13]. These include radiometric calibration [14], non-uniformity correction (NUC) [15,16], and the contribution of environmental radiance to the observed scene’s radiance. Among these different errors, the assessment of emissivity is considered the primary source of error.

According to the work of Monchau and Hameury [17], spectral directional emissivity appears to be of appropriate definition for thermography. It enables the definition of emissivity in the observed scene within the spectral band of the infrared camera. However, determining the emissivity of the observed scene is challenging because of its multifactorial dependence. For a given material, the viewed emissivity depends on various factors, including the spectral range of the camera [18], the absolute temperature of the material [19], the surface properties and preparation of the observed object (such as color, roughness, reflectance, uniformity) [20], and the incidence angle between the observed object and the camera lens [21].

Emissivity can be measured using calorimetric or radiometric methods. In radiometric methods, both direct (using the ratio between the black body and sample radiance) and indirect (commonly employing Kirchhoff’s law) approaches may be utilized. Furthermore, there are two main methods using infrared cameras for measuring emissivity. The first method involves observing a scene with a known temperature to deduce emissivity, while the second method measures the reflectivity of the scene to calculate emissivity. Another approach involves using a bi-chromatic pyrometer to isolate two spectral ranges and determine emissivity [22,23,24].

Despite the various means of measuring emissivity, such as optical pyrometers, there is currently no method available to determine emissivity during infrared camera acquisition. Moreover, none of the existing methods can provide a pixel-wise mapping (i.e., local) of emissivity; instead, they typically provide an averaged value (i.e., global) of emissivity. This study proposes a method that utilizes a multispectral infrared camera to obtain pixel-wise emissivity of an observed scene during camera recording, without the need for additional means of measure.

## 2. Experimental Setup

The aim of this study is to obtain the emissivity of a sample observed by an infrared camera. To this end, the camera Telops MS-M3k (TELOPS, Quebec, Canada) has been used. This infrared camera has an Insb (Indium Atimonide) sensor operating within the spectral range of 1.50 to 5.50 µm, with a resolution of 320 × 256 pixels, a pitch of 30 µm, and an indicated Noise Equivalent Temperature Difference (NETD) of 25 mK. A specificity of this camera is that it is equipped with a filter wheel controllable rotational speed, with the possibility to set independent integration times for each filter. For this study, the filter wheel was set with two different filters: a broadband filter (BB) and a spectral filter (SF), arranged alternatively on the filter wheel (a schema of the filter wheel configuration is presented in Table 1). The broadband and the spectral filters have respective spectral ranges of 3.11–5.50 µm and 5.08–5.50 µm, and allow observation of radiometric temperatures from 0 °C to 240 °C and 100 °C to 400 °C, respectively.

The optical component chosen for this setup is an ×4 camera lens, providing a spatial resolution of 7.5 µm.pixel^−1^. It is important to note that the current configuration of the filter wheel may not be optimized for measuring the emissivity of an observed scene, as the spectral range of each filter is relatively large. Ideally, narrow and closely spaced spectral ranges would be more suitable to achieve the best accuracy. However, the chosen filter wheel configuration allows for observation of a wide temperature range which is necessary for the machining application in our laboratory, as well as for conducting several experimental tests. Furthermore, the proposed method is independent of the chosen filter ranges. Nevertheless, for specific applications aiming for better metrological performance (such as a particular temperature range), selecting filter pairs with narrower and closer spectral ranges would be beneficial.

To enhance accuracy, an independent radiometric calibration was performed for each pixel and for each filter of the filter wheel, as described by [25]. This calibration was carried out using an Ametek Landcal P1200B black body source (AMETEK Land, Berwyn, PA, USA). With the current configuration of the filter wheel, the infrared camera used in the study can be considered a multispectral infrared camera. The camera settings are summarized in Table 1.

**Figure 1:**
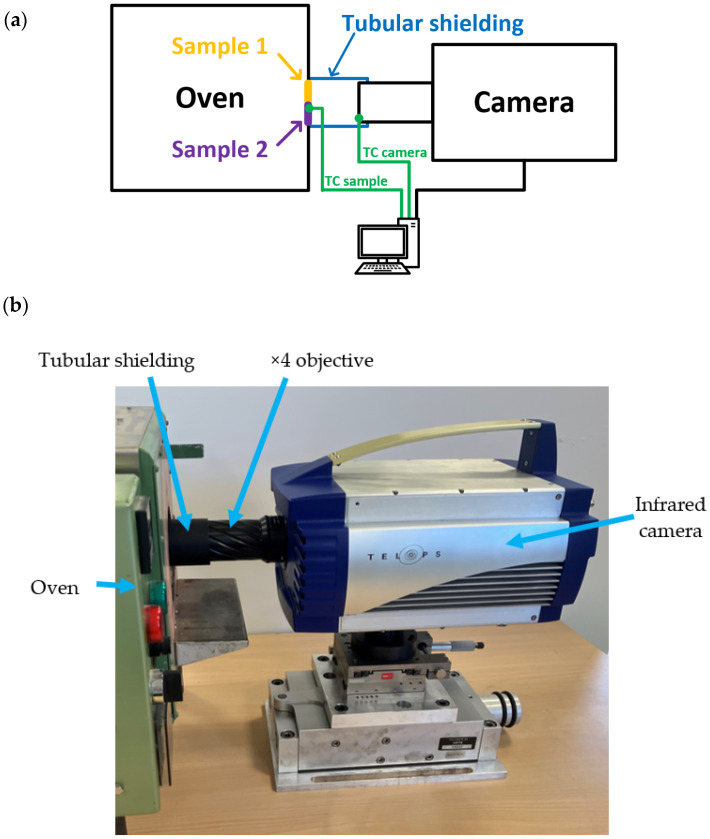
Experimental setup: principle schema (**a**), global view (**b**).

The experimental setup, designed to obtain the local emissivity of the two observed samples using a multispectral infrared camera, is illustrated in the Figure 1. In this study, Inconel 718 was chosen as the material of interest owing to extensive prior research and the potential for exhibiting different global emissivities based on surface preparation. Additionally, examining the microscale level where the microstructure is exposed provides an opportunity to observe local variations in emissivity.

The observed scene consisted of two Inconel 718 samples. The first sample was an Inconel 718 wire-cut using Electrical Discharge Machining (EDM), while the second sample was the same Inconel 718 material polished using an automatic polisher with a 1 µm grit and a chemical etching using the Adler reagent. By placing these two samples side by side, distinct differences in global emissivity were achieved within the same scene. Both samples were heated and maintained at a temperature of 200 °C using an oven. This temperature was selected to ensure proper observation conditions for both samples with the broadband and spectral filters. For the polished sample, the radiometric temperature was approximately 105 °C when observed through the spectral filter, and for the wire-cut EDM sample, it was around 150 °C with the broadband filter. Therefore, setting the absolute temperature to 200 °C allowed for accurate observations of both samples under appropriate conditions. To ensure correct pixel well-filling during measurements, integration times of 25 µs for the broadband filter and 75 µs for the spectral filter were chosen, resulting in a pixel load of approximately 90% for the wire-cut EDM sample and 50% for the polished sample. Additionally, an acquisition frame rate of 800 Hz, which corresponds to the maximum available frame rate because of the rotation speed of the filter wheel, was selected. This high frame rate ensures that temperature variations in the observed scene (e.g., due to oven temperature regulation) between consecutive pictures are insignificant.

The camera was positioned in front of the scene to be viewed, and a matte black tubular shielding was added along the optical path between the lens and the samples. This carter effectively absorbs the majority of the surrounding radiance, reducing the impact of environmental radiance on the measurements. Furthermore, two thermocouples were placed to obtain local temperatures: one on the surface of the observed samples and the other next to the camera lens inside the surrounding carter.

## 3. Pixel-wise Emissivity Measurement Method

### 3.1. Hypothesis

In all the present study, the link between the radiance and the temperature will be established using Planck’s law, shown in Equation (1):

(1) $$\begin{array}{c} Rad{\left(T_{BBS}\right)}_{Num\_F}=\int_{\lambda_{1}{}_{Num\_F}}^{\lambda_{2}{}_{Num\_F}}\frac{2\;h\;c^{2}\;\lambda^{-5}}{\exp\left(\frac{h\;c\;}{\lambda \;k\;T}\right)-1}\;d\lambda \; \end{array}$$

with Num_F being the position of the filter on the filter wheel, Rad as the observed radiance, T as the absolute temperature of the black body source in kelvin, h as Planck’s constant (h = 6.6226176 × 10^−34^ [J.s]), k as the Boltzmann constant (k = 1.380662 × 10^−23^ [J.K^−1^]), c as the celerity of the light (c = 2.998 × 10^8^ [m.s^−1^]), and λ as the considered wavelength in meters. In all the following, the wavelength interval of integration of Planck’s law ($\lambda_{1},\;\lambda_{2}$) corresponds to the spectral range of the filter considered (3.11–5.50 µm for the broadband filter, and 5.08–5.50 µm for the spectral filter). Moreover, given that this integral has no analytical solution, it was calculated numerically by the trapezoidal rule method, with wavelength discretization steps of 3.9 × 10^−4^ µm for each filter.

#### 3.1.1. Influence of the Environment on the Measurement

In this study, it was assumed that atmospheric transmission has a negligible impact on temperature and emissivity measurements, especially when compared to other sources of errors. This assumption is based on the works of Monchau [26] and Pajani [27]. Considering the experimental conditions of this study, where the lens used has a working distance of 33 mm, it was estimated that this hypothesis introduces an error in the measured radiometric temperature of less than 1 K. It is important to mention that this assumption holds true because the utilized objective has a short working distance. When using objectives with a macro range working distance (usually distance in meters), this assumption no longer holds true as in this case; therefore, the proposed method may not be applicable. As previously mentioned, the observed scene consisted of two samples of Inconel 718, each with a thickness of 2 mm. In this context, it was assumed that the transmission ratio through the samples was zero, meaning that no radiant energy from the surrounding environment passes through the samples. Therefore, the fraction of the radiant energy from the environment captured by the camera lens can be defined using the Equation (2):

(2) $$\begin{array}{c} R_{A}=1-\varepsilon_{0} \end{array}$$

with $R_{A}$ as the fraction of the environment radiant energy, and $\varepsilon_{0}$ as the emissivity of the observed scene. Furthermore, it was assumed that the emissivity of the environment’s radiant energy was 1. To uphold this assumption, a carter with a high emissivity inner coating was utilized between the observed samples and the camera lens, as described in the experimental setup (Section 2, Figure 1). By using the suggested approach, the impact of the environment on radiance is evaluated, and it can be considered to have no further influence on the calculated absolute temperature and emissivity of the samples. Taking into account all the aforementioned assumptions and referring to the findings of Li et al. [28], the expression for the observed radiance can be defined as the Equation (3):

(3) $$\begin{array}{c} Rad{\left(Num_{im,X,Y}\right)}_{Num\_F}=\epsilon_{\lambda_{1},\lambda_{2}}*\int_{\lambda_{1}{}_{Num\_F}}^{\lambda_{2}{}_{Num\_F}}\frac{2\;h\;c^{2}\;\lambda^{-5}}{\exp\left(\frac{h\;c\;}{\lambda \;k\;T_{Num_{im},X,Y}}\right)-1}\;d\lambda +\left(1-\epsilon_{\lambda_{1},\lambda_{2}}\;\right)\;*\int_{\lambda_{1}{}_{Num\_F}}^{\lambda_{2}{}_{Num\_F}}\frac{2\;h\;c^{2}\;\lambda^{-5}}{\exp\left(\frac{h\;c\;}{\lambda \;k\;T_{env}}\right)-1}\;d\lambda \; \end{array}$$

with $Num_{im,X,Y}$ as the number of the image im and the pixel coordinates X,Y, $T_{Num_{im},X,Y}$ as the absolute temperature of the image im and the pixel coordinates X,Y, and $T_{env}$ as the environment temperature.

#### 3.1.2. Constant Emissivity on Spectral Ranges of the Two Filters

In infrared thermography, when using a specific filter, the camera observes a scene within a defined spectral range. To obtain an absolute temperature measurement, it is necessary to assume that the emissivity of the observed scene remains constant over the spectral range of the filter or to use the mean emissivity within the operating spectral range. The objective of this study is to obtain the emissivity of the observed scene by capturing two different pictures using two different spectral filters: the broadband filter and the spectral filter. Thus, it is crucial to hypothesize that the emissivity of the observed scene remains constant within the spectral range covered by both filters. The closer the spectral ranges of the two pictures are, the more consistent the assumption of constant emissivity over both ranges becomes.

In this study, the broadband filter results in a spectral range of 3.11–5.50 µm, while the spectral filter produces a range of 5.08–5.50 µm. Therefore, for the remainder of this study, the hypothesis is made that the emissivity of the observed scene is constant within the merged spectral range of 3.11–5.50 µm.

**Figure 2:**
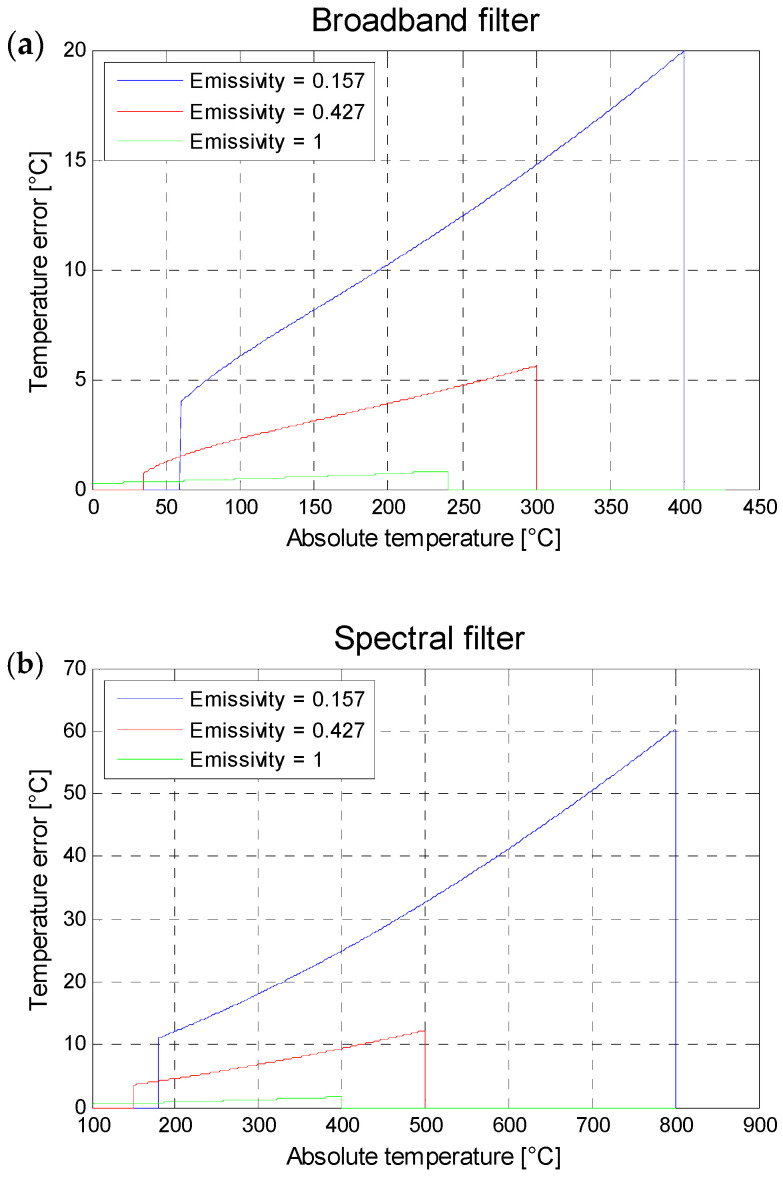
Temperature error implied by an error over the emissivity of 0.025, as a function of the absolute temperature of the observed scene and for three different values of emissivity for the following filters (**a**) broadband filter, and (**b**) the spectral filter.

The tested material in this study is an Inconel 718. According to the results reported by Del Campo [21], for Inconel 718 and the spectral range of 3.11–5.50 µm, the highest fluctuation in emissivity is observed for a surface that has been wire-cut using EDM. The study indicates that the emissivity changes from 0.75 at 3.11 µm to 0.70 at 5.50 µm, resulting in a variation of emissivity of 0.05 within the spectral range of 3.11–5.50 µm. This allows us to consider the emissivity as constant for this material and within the spectral range used in the experimental setup of this study. In a first approximation, it can be assumed that the camera captures the mean radiance within its spectral range. Therefore, a variation of 0.05 in emissivity corresponds to a mean error of 0.025 in emissivity. To assess the impact of an emissivity error of 0.025 on the calculated absolute temperature, simulations based on the radiometric model described by Equation (3) were conducted. Table 2 summarizes the input parameters of the radiometric model, and Figure 2 illustrates the error in absolute temperature measurement as a function of the actual absolute temperature of the scene for an emissivity error of 0.025.

In summary, for an absolute temperature of the samples at 200 °C (which is the chosen temperature for the experimental setup described in Section 2), an error of 0.025 in the emissivity of the observed scene (specifically, the surfaces of the Inconel 718 samples) would result in an error in the measured absolute temperature by the infrared camera of less than 10 °C. This level of error appears to be acceptable for infrared thermography applications.

### 3.2. Problem Setting

Based on the previous part and more particularly on Equation (3), the radiance of the observed scene is expressed from the emissivity of the sample, the sample absolute temperature, and the temperature of the environment; all of these are unknowns to assess. Conceptually, the observed radiance is expressed with 3 unknowns and the filter wheel of the camera is composed of 2 different filters. Therefore, the equations system is wrongly set (underdetermined problem setting) and not solvable in this configuration. To enhance the number of available equations and make the system resolvable, it was decided to observe, in the same scene, 2 different samples with different surface preparations (and, therefore, different emissivity). The first sample is an Inconel 718 wire-cut by EDM, and the second is polished and etched by an Adler solution. Thanks to this tip, and by considering that the absolute temperature and the incident environment radiance the same for the two samples, the system of equations was then composed of 4 equations (1 equation for each sample and for each filter) with 4 total unknowns. Then, the system was resolvable (determined problem setting), and to resume, the unknown parameters to be determined were as follows: the emissivity of each sample, the absolute temperature of the 2 samples, and the radiance of the environment. Equation (4) sums up the equations system used to obtain local emissivities (i.e., for every pixel of the captured frame) of the observed scene.

(4) $$\{\begin{array}{l} Rad{\left(Sample1_{Num\_im,X,Y}\right)}_{BB}=\epsilon_{Sample1,X,Y}*\int_{\lambda_{1}{}_{BB}}^{\lambda_{2}{}_{BB}}\frac{2\;h\;c^{2}\;\lambda^{-5}}{\exp\left(\frac{h\;c\;}{\lambda \;k\;T_{Num_{im},X,Y}}\right)-1}\;d\lambda +\left(1-\epsilon_{Sample1,X,Y\;}\;\right)\;*\int_{\lambda_{1}{}_{BB}}^{\lambda_{2}{}_{BB}}\frac{2\;h\;c^{2}\;\lambda^{-5}}{\exp\left(\frac{h\;c\;}{\lambda \;k\;T_{env}}\right)-1}\;d\lambda \\ Rad{\left(Sample1_{Num\_im,X,Y}\right)}_{SF}=\epsilon_{Sample1,X,Y}*\int_{\lambda_{1}{}_{SF}}^{\lambda_{2}{}_{SF}}\frac{2\;h\;c^{2}\;\lambda^{-5}}{\exp\left(\frac{h\;c\;}{\lambda \;k\;T_{Num_{im},X,Y}}\right)-1}\;d\lambda +\left(1-\epsilon_{Sample1,X,Y\;}\;\right)\;*\int_{\lambda_{1}{}_{SF}}^{\lambda_{2}{}_{SF}}\frac{2\;h\;c^{2}\;\lambda^{-5}}{\exp\left(\frac{h\;c\;}{\lambda \;k\;T_{env}}\right)-1}\;d\lambda \\ Rad{\left(Sample2_{Num\_im,X,Y}\right)}_{BB}=\epsilon_{Sample2,X,Y}*\int_{\lambda_{1}{}_{BB}}^{\lambda_{2}{}_{BB}}\frac{2\;h\;c^{2}\;\lambda^{-5}}{\exp\left(\frac{h\;c\;}{\lambda \;k\;T_{Num_{im},X,Y}}\right)-1}\;d\lambda +\left(1-\epsilon_{Sample2,X,Y\;}\;\right)\;*\int_{\lambda_{1}{}_{BB}}^{\lambda_{2}{}_{BB}}\frac{2\;h\;c^{2}\;\lambda^{-5}}{\exp\left(\frac{h\;c\;}{\lambda \;k\;T_{env}}\right)-1}\;d\lambda \\ Rad{\left(Sample2_{Num\_im,X,Y}\right)}_{SF}=\epsilon_{Sample2,X,Y}*\int_{\lambda_{1}{}_{SF}}^{\lambda_{2}{}_{SF}}\frac{2\;h\;c^{2}\;\lambda^{-5}}{\exp\left(\frac{h\;c\;}{\lambda \;k\;T_{Num_{im},X,Y}}\right)-1}\;d\lambda +\left(1-\epsilon_{Sample2,X,Y\;}\;\right)\;*\int_{\lambda_{1}{}_{SF}}^{\lambda_{2}{}_{SF}}\frac{2\;h\;c^{2}\;\lambda^{-5}}{\exp\left(\frac{h\;c\;}{\lambda \;k\;T_{env}}\right)-1}\;d\lambda \end{array}$$

In order to solve the equations system described in Equation (4), a possibility is to numerically vary unknown parameters with a view to optimizing each value through the minimization of a dedicated criterion. Given that each pixel of the sensor observes a specific zone of the scene, they all should receive different values. Therefore, a pixel-wise method has been chosen for the program (from the calibration method to the calculation of emissivity). For this, the following procedure was implemented in a Matlab R2022b program involving two interlocked loops:

- At first, digitals levels of each pixel of the infrared camera sensor have to be converted into radiance. For this, the radiometric calibration of the infrared camera and Planck’s law are necessaries;
- Secondly, for a given environment temperature and for each pixel, the emissivity values that minimize the absolute temperature difference between the broadband filter and the spectral filter have to be determined by the first numerical loop. The term of emissivity has been chosen to vary from 0 to 1 by steps of 0.001 and, for each iteration, a test is done to check whether the value of the emissivity minimizes the criterion. The Algorithm 1 presents this subroutine;
- Thirdly, in a second numerical loop, the environment temperature is identified by minimizing the mean absolute temperature difference between the two samples, as explained in the Algorithm 1. Next, for each term of environment temperature, a test is done to check whether the value minimizes the absolute temperature difference between the wire-cut by EDM and the polished sample. Algorithm 2 presents this routine.
- At the end, the program outputs the following: the local values of the emissivity and the local absolute temperature of each pixel, and the global radiometric temperature of the environment, all of them being the result of the minimization of the absolute temperature difference between the two filters and the two samples.

**Algorithm 1:** Emissivity calculation**Input:** Radiance of the observed scene, *T_env_*
  **Set** the step on emissivity: step_e_
  **For** e = 0: step_e_: 1  **Calculate** *T_BB_* and *T_FS_*$\;Rad{\left(Num_{Im}\right)}_{X,Y}=\epsilon_{\lambda_{1},\lambda_{2}}\times \int_{\lambda_{1\mathrm{Num}\_F}}^{\lambda_{2\mathrm{Num}\_F}}\frac{2\;h\;c^{2}\;\lambda^{-5}}{\exp\left(\frac{h\;c\;}{\lambda \;k\;T_{Num_{im},X,Y}}\right)-1}\;d\lambda +\left(1-\epsilon_{\lambda_{1},\lambda_{2}}\;\right)\times \int_{\lambda_{1\mathrm{Num}\_F}}^{\lambda_{2\mathrm{Num}\_F}}\frac{2\;h\;c^{2}\;\lambda^{-5}}{\exp\left(\frac{h\;c\;}{\lambda \;k\;T_{Env}}\right)-1}\;d\lambda$  **If** e minimizes abs(*T_BB_* − *T_FS_*)  e_f_ = e  *T_f_* = (*T_BB_* − *T_FS_*)/2  **End If**
  **End For****Output:** e_f_ and *T_f_*

**Algorithm 2:** Identification of the calibration matrixes**Input:** Camera data of the observed scene
  **Open** camera files 
  **Convert** raw data in radiance
  **For** $env=env_{min}\;:\;step_{env}\;:\;env_{max}$  Run Algorithm 1$\;T_{EDM\;sample}=\frac{1}{n_{EDM}}\;\sum_{x_{EDM}\;;\;x_{EDM}}^{n_{EDM}}{\left(T_{f}\right)}_{x_{EDM}\;;\;x_{EDM}}$$\;T_{polished\;sample}=\frac{1}{n_{polished}}\;\sum_{x_{polished}\;;\;x_{polished}}^{n_{polished}}{\left(T_{f}\right)}_{x_{polished}\;;\;x_{polished}}$  **If** env minimizes $abs\;\left(T_{EDM\;sample}-T_{polished\;sample}\right)$$\;T_{env}=env$  **End If**  **End For****Output:** e_f_, *T_f_*, *T_env_*

The present approach for the resolution of Equation (4) is based on 2 samples, which introduce 2 significant emissivity values. Practically, the problem could be solved by considering one sample, i.e., formulating equations at different pixels of a unique sample having different emissivities.

## 4. Experimental Results and Discussion

The experimental setup developed to determine the emissivity and the absolute temperature of two samples in Inconel 718, is described in Section 2. To this end, the camera Telops MS-M3k was used, with a ×4 camera lens, which provides a spatial resolution of 7.5 µm.pixel^−1^ at a fixed working distance of 33 mm. It is important to note that the current configuration of the filter wheel may not be optimized to measure the emissivity of an observed scene, as the spectral range of each filter is relatively large. Ideally, narrow and closely spaced spectral ranges would be more suitable for achieving the best accuracy. However, the chosen filter wheel configuration allows for observation of a wide temperature range, which is necessary for the machining application in our laboratory, as well as for conducting several experimental tests. Furthermore, the proposed method is independent of the chosen filter ranges. Nevertheless, for specific applications aiming for better metrological performance (such as a particular temperature range), selecting filter pairs with narrower and closer spectral ranges would be beneficial.

To enhance accuracy, an independent radiometric calibration was performed for each pixel and for each filter of the filter wheel. This calibration was carried out using an Ametek Landcal P1200B black body source. With the current configuration of the filter wheel, the infrared camera used in the study can be considered a multispectral infrared camera. The camera settings are summarized in Table 1 and the Table 3 presents input parameters of the algorithm described in the previous section.

**Figure 3:**
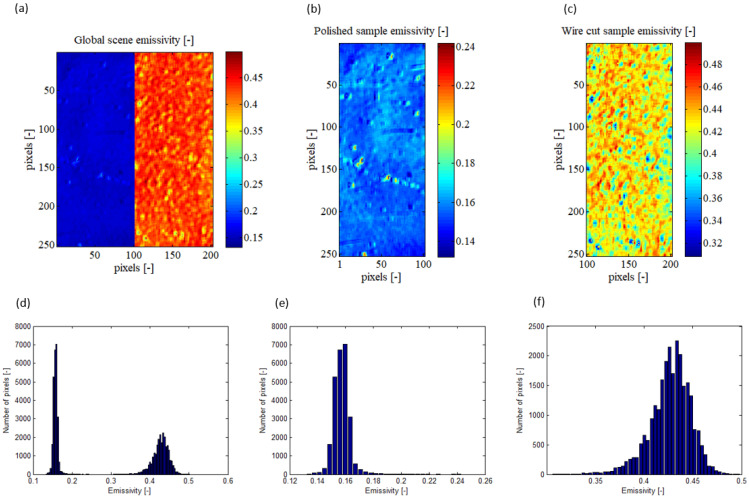
Calculated emissivity presented on XY sample map and its pixel repartition histogram for the global observed scene (**a**,**d**), for the polished sample (**b**,**e**), and for the EDM wire-cut sample (**c**,**f**).

As outputs, the algorithm gives the absolute temperatures and the emissivity of the observed scene for each pixel of the sensor. The Figure 3 shows the emissivity calculated by the algorithm for the global observed scene and for each sample.

Firstly, in the Figure 3a, it is observed that the mapping is created from a resolution of 200 × 256 pixels^2^, whereas the Telops MS-M3k camera has a native resolution of 320 × 256 pixels^2^. This is due to the numerical deletion of the interface region between the two samples. This decision was made because this region exhibited singular results with high variability in emissivity. The irregularities in this interface region, such as chamfers, interface quality, and local variations in emissivity due to surface heterogeneity, led to inconsistent results. Therefore, it is concluded that this region is not representative of the observed samples in the context of this study.

**Figure 4:**
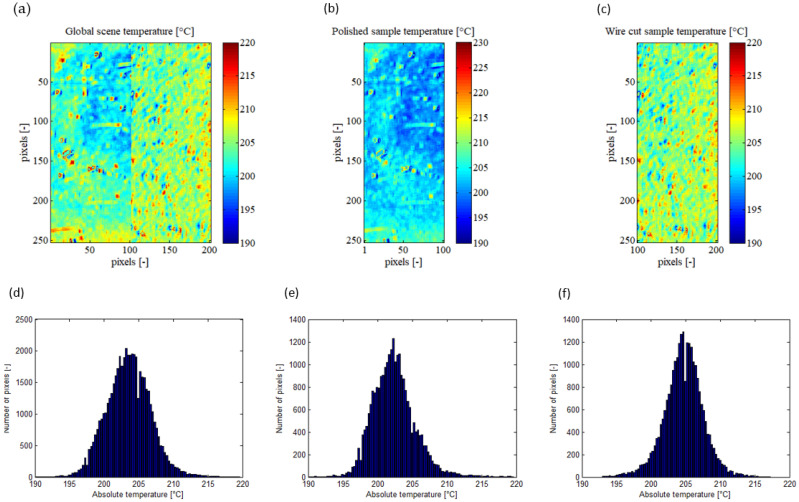
Absolute temperature of the scene calculated by the algorithm, presented on XY sample map and its pixel repartition histogram for the global observed scene (**a**,**d**), for the polished sample (**b**,**e**), and for the wire-cut sample (**c**,**f**).

The histograms presented in the Figure 3e,f depict the distribution of emissivity for the polished and wire-cut samples, respectively (as shown in the Figure 4b,c). It can be observed that the polished sample has a mean emissivity of 0.1572 with a standard deviation of 0.0059, while the wire-cut sample has a mean emissivity of 0.4270 with a standard deviation of 0.0211. These results indicate that the observed scene is indeed composed of heterogeneous emissivity, underscoring the significance of employing a pixel-wise method rather than a more global measurement approach. Emissivity is a critical parameter in infrared thermography and its heterogeneity can introduce substantial errors.

Figure 4 illustrates the absolute temperatures calculated by the emissivity determination algorithm for the overall scene and each sample. From Figure 4d, it can be observed that the algorithm determined a mean absolute temperature of 203.53 °C for the entire observed scene, with a standard deviation of 3.21 °C. All temperatures fell within the range of 190 °C to 220 °C. During recording with the infrared camera, the thermocouple placed on the samples measured a mean local temperature of 200.0 ± 0.5 °C. Thus, the average deviation in absolute temperature between the thermocouple and the infrared camera was approximately 3.5 °C. It is important to note that the thermocouple and the developed method are independent means of temperature measurement. Given this result, it appears that the algorithm is capable of reliably calculating the absolute temperature and emissivity of an observed scene composed of heterogeneous emissivities using this pixel-wise method.

Based on the histograms presented in Figure 3e,f, the mean absolute temperatures measured by the infrared camera for the polished and wire-cut samples are 202.41 °C and 204.67 °C, respectively. The standard deviation of the measured absolute temperature is 3.45 °C for the polished sample and 2.94 °C for the wire-cut sample. Interestingly, despite the wire-cut sample having a higher standard deviation in emissivity compared to the polished sample, its standard deviation in absolute temperature is smaller. This suggests that the emissivity of the wire-cut sample is more heterogeneous than that of the polished sample. Nonetheless, the algorithm is still able to accurately calculate the heterogeneous emissivity and converge towards a low standard deviation in absolute temperature.

## 5. Pixel-wise Emissivity Assessment toward Other Methods

Compared to actual different ways to get emissivity, the proposed method is the closest to a bi-chromatics pyrometer. Using this technology, the two spectral ranges are significantly narrower than the ones used with the configuration of the infrared camera in the present study. Therefore, the necessary hypothesis, which supposes that the emissivity of the observed scene is constant on the spectral ranges of the two filters, implies a lower error with the pyrometer than with this camera. Nevertheless, the developed method offers distinct advantages over a bi-chromatics pyrometer. Firstly, with the proposed method, it is possible to calculate the environment radiance of the observed scene, which enhances the accuracy of the calculated emissivity and allows for better control and validation of the results using the measured radiance environment value. In contrast, a bi-chromatics pyrometer typically omits or requires the user to estimate the environment radiance using other measurement methods. Secondly, while a bi-chromatics pyrometer provides a single global averaged measurement (equivalent to one pixel), the infrared camera discretizes the region of interest into multiple pixels on the sensor. This is particularly advantageous when dealing with scenes composed of heterogeneous emissivity.

**Figure 5:**
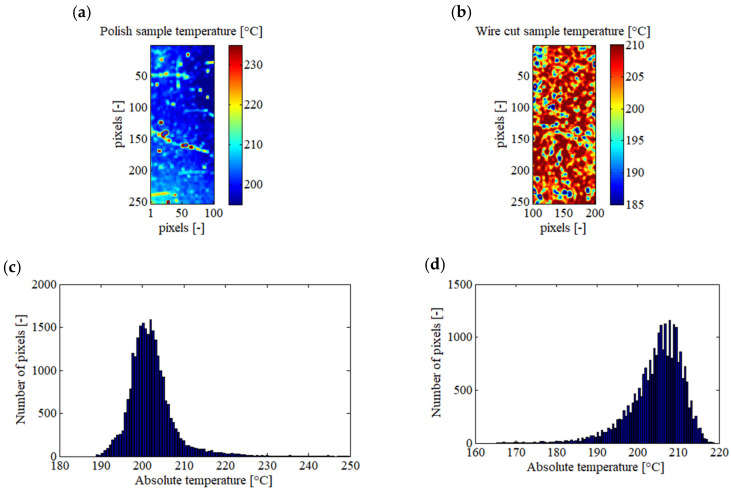
Absolute temperatures with the hypothesis of homogeneous emissivity for each sample, presented as images and histograms. The polished sample with an emissivity of 0.1572 (**a**,**c**), and wire-cut sample with an emissivity of 0.4270 (**b**,**d**).

To demonstrate a potential result that could have been obtained with a bi-chromatics pyrometer, the absolute temperatures of each sample using their respective mean emissivity (0.1572 for the polished sample and 0.4270 for the wire-cut sample) were calculated. The results are depicted in Figure 5.

Table 4 compares results obtained from the proposed method toward equivalent bi-chromatics optical pyrometers. Concerning the polished sample, by considering a constant emissivity, a mean absolute temperature of 202.18 °C with a standard deviation of 5.87 °C is calculated and all the temperatures fall within the interval [188; 249] °C. Whereas, for the same sample, using the proposed method, a mean absolute temperature of 202.41 °C with a standard deviation of 3.45 °C was measured and all the measurements fall within the interval [190; 220] °C. In addition, in Figure 5a, it is possible to see lines with higher temperatures, which are not present in Figure 4b. In practice, these lines in Figure 5a are caused by scratches on the sample, involving local increases in emissivity. Therefore, the reduction of the presence of these lines in Figure 4b is an example of the advantages of this method compared to choosing a constant emissivity for the entire sample. In the case of the wire-cut sample and by considering a constant emissivity, a mean absolute temperature of 204.82 °C with a standard deviation of 6.57 °C is calculated and all the temperatures fall within the interval [165; 219] °C. Whereas, with the proposed pixel-wise method, a mean absolute temperature of 204.67 °C with a standard deviation of 2.94 °C was measured and all the measurements fall within the interval [190; 220] °C. In the end, it seems that the mean and the standard deviation of the absolute temperature calculated by the proposed method could be more accurate than results obtained using the bi-chromatics pyrometer. However, thanks to this method, all the pixels are restrained in an acceptable temperature interval, even in presence of local variation of emissivity; this is not the case with the bi-chromatics pyrometer.

## 6. Conclusions

This study presents a method for directly calculating pixel-wise emissivity of a scene observed by a multispectral infrared camera. This approach is based on the principle of a bi-chromatics pyrometer but implemented using a multispectral camera equipped with at least two filters with different spectral ranges. By discretizing the observed scene based on the sensor’s resolution, emissivity values can be obtained for each pixel. Furthermore, this method enables an accurate assessment of the environment radiance.

The main advantage of this method is that it allows for the calculation of pixel-wise emissivity during the recording of infrared camera images, without the need for additional materials or experiments. As a result, it becomes possible to observe scenes with heterogeneous emissivity. Additionally, since the emissivity of each pixel is measured for each frame, if the emissivity changes during the recording, the algorithm can update the emissivity values for each camera frame. This is a significant advantage compared to other methods. Moreover, the chosen filters mounted in the rotative filter wheel enable the measurement of pixel-wise emissivities and their corresponding absolute temperatures of the observed scene during quasi-static experimental tests, with high measurement performance. However, it should be noted that infrared cameras need to observe the entire phenomenon being studied, which often requires a wide range of temperature capabilities. As a result, the choice of the two filters used in this study is limited, as each filter must be able to observe the full temperature range of the studied phenomenon. Therefore, compared to a traditional bi-chromatics pyrometer operating within its optimal range, the proposed method may yield less accurate results. To enhance this study, it would be interesting to determine, based on the temperature range of the observed scene, the set of filters that optimize the absolute temperature measurements.

## Figures and Tables

**Table 1:** Infrared camera features and setting.

Parameters	Values	
Insb sensor resolution	320 × 256 [pixel^2^]	
Pitch	30 [µm]	
NETD	25 mK	
Lens magnification	×4	
Working distance	33 [mm]	
Depth of field	1 [mm]	
Spatial resolution	7.5 [µm/pixel]	
Observation window	2.40 × 1.92 [mm^2^]	
Frame rate	800 [Hz]	
Filter wheel configuration	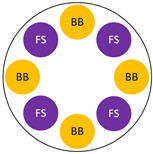	
Type of filter	Broadband (BB)	Spectral (FS)
Spectral range	3.11–5.50 [µm]	5.08–5.50 [µm]
Radiometric temperatures range	0–240 [°C]	100–400 [°C]
Integration time	25 [µs]	75 [µs]

**Table 2:** Input parameters of the radiometric model.

Parameters	Values
Spectral range of the broadband filter: λ_1_–λ_2_	3.11 × 10^−6^–5.50 × 10^−6^ [m]
Spectral range of the spectral filter: λ_1_–λ_2_	5.08 × 10^−6^–5.50 × 10^−6^ [m]
Mean error on emissivity	0.025 [-]
Environment temperature: *T_env_*	20 [°C]
Step of wavelength for the calculation of the Planck’s integral	3.90 × 10^−10^ [m]
Step of temperature for the calculation of the Planck’s integral	0.001 [K]

**Table 3:** Input parameters of the algorithm that determined the emissivity, the absolute temperature of the observed scene, and the radiometric temperature of the environment.

Parameters	Values
Step of wavelength for the calculation of the Planck’s integral	3.90 × 10^−10^ [m]
Step of temperature for the calculation of the Planck’s integral	0.1 [K]
Step of emissivity in the first loop	0.001 [-]
Step of environment temperature in the second loop	1 [K]

**Table 4:** Confrontation of the present pixel-wise emissivity assessment method with an equivalent bi-chromatics optical pyrometer.

Source	Emissivity [-] (Average ± Standard Deviation)		Absolute Temperature [°C] (Average ± Standard Deviation) [Min; Max]		Temperature Thermocouple Type K in °C
	Polished/Adler	Wire-cut	Polished/Adler	Wire-cut	
Equivalent bi-chromatic pyrometer	0.1572	0.4270	202.18 ± 5.87 [188; 249]	202.41 ± 3.45 [190; 220]	200
Infrared camera with 2 filters	0.1572 ± 0.0059	0.4270 ± 0.0211	204.82 ± 6.57 [165; 219]	204.67 ± 2.94 [190; 220]	

## Data Availability

The data presented in this study are available on request from the corresponding author.
